# Psycho-Emotional Factors Associated with Depressive Symptoms during Lockdown Due to the COVID-19 Pandemic in the Mexican Population

**DOI:** 10.3390/ijerph20054331

**Published:** 2023-02-28

**Authors:** Nora A. Martínez-Vélez, Miriam Arroyo-Belmonte, Marcela Tiburcio, Guillermina Natera-Rey, Morise Fernández-Torres, Graciela Y. Sánchez-Hernández

**Affiliations:** Department of Social Sciences in Health, Direction of Epidemiological and Psychosocial Research, Ramón de la Fuente Muñiz National Institute of Psychiatry, Calzada Mexico-Xochimilco 101, San Lorenzo Huipulco, Tlalpan, Mexico City 14370, Mexico

**Keywords:** mental health, depressive symptomatology, COVID-19

## Abstract

The COVID-19 pandemic has had a significant impact on mental health, leading to the increase of depressive symptoms. Identifying these symptoms and the factors associated with them in women and men will allow us to understand possible mechanisms of action and develop more specific interventions. An online survey was conducted from 1 May to 30 June 2020 using snowball sampling; the final sample comprised 4122 adult inhabitants of Mexico; 35% of the total sample displayed moderate-to-severe depressive symptoms, with a greater proportion of depression being among female respondents. A logistic regression analysis revealed that individuals under 30 years of age, those with high levels of stress due to social distancing, those with negative emotions, and those who reported a significant impact of the pandemic on their lives have a higher risk of depression. Women with a history of mental health treatment and men with a history of chronic disease were also more likely to experience depressive symptoms. Social environment and sex are factors that intervene in the development of depressive symptoms, meaning that appropriate early identification and intervention models should be designed for the care of men and women in highly disruptive situations such as the recent pandemic.

## 1. Introduction

The public health emergency caused by COVID-19 began in December 2019, with the first cases in Mexico being identified in February 2020 and the declaration of a national emergency in the country at the end of March 2020 [1]. This emergency posed complex challenges for the general population as, in addition to the obvious consequences for physical health, it affected the mental health of men and women [2]. Much of the literature published to date has identified an increase in moderate or severe symptoms of acute stress, anxiety, and depression [3,4,5,6,7,8,9]. Since the start of the pandemic, there has been a worldwide increase of 27.6% in depressive disorders, with women and young people being the most affected [10]. Mexico has been no exception: an online survey of medical students conducted from April to December 2020 found that the prevalence of depressive symptoms increased during that period from 19.84% to 40.8% [11].

Few studies, however, have analyzed the factors behind this increase. Stress is well-known as a trigger of depressive reactions, fear, and anxiety [12,13,14,15,16]. Stressors related to previous natural disasters and accidents have been linked to declines in mental health not just during these events, but for many months or years after the events [17,18].

The stress generation model of depression (SGMD) [19,20] has substantially contributed to an advanced understanding of the relationship between stress and depression and of the factors and mechanisms involved in its persistence and recurrence; it allows us to understand that not all domains of stressful life events have an equal effect on people’s health. This model differentiates the impact of factors associated with the discord in people’s interpersonal relationships and the factors of their context (such as financial stress, academic or work difficulties, and poor health) in the shaping of depression [12,19,20]. The COVID-19 pandemic affected interpersonal relationships through restrictions on social interactions [21], and it has also generated contextual changes such as disruptions in employment, personal finances, and work–life balance [22,23].

However, we do not know if these factors have affected women and men equally or if they have had a differentiated impact on this increase in depression. Studies carried out at the start of the pandemic have found that being female, younger (particularly under 35 years of age), and having less education or financial resources were variables that were associated with the presence of depressive symptoms [9,16,24]. Other studies, however, suggest that various factors have been associated with a higher risk of depression during the COVID-19 pandemic. Early in the pandemic, pre-existing mental health conditions, living alone, and marital status were associated with elevated levels of anxiety and/or depression [25]. Physical health conditions, being in close contact with people with COVID-19, mental comorbidity, coping styles, stigmas, psychosocial support, personal protection measures, risk of contracting COVID-19, and concerns that a family member would be infected were also associated with depression [2,26].

In addition to these contextual stress factors, psycho-emotional factors also play a fundamental role in the configuration of depressive symptomatology. A pandemic triggers an emotional response [27] that can range from risk denial to high levels of fear and anxiety [28]. Some studies have suggested that women tend to be more worried, anxious, scared, sad, and angry than men [29,30]. The SGMD has also recognized the important role of emotion as an associated factor, which is usually conceptualized in broad categories such as negative versus positive emotions [31]. For example, it has been observed that positive affect may facilitate resilience in the presence of stressors and reduce vulnerability to mental health disorders [32].

Although there is evidence of differences between women and men in the stress and worry experienced during the pandemic, the contextual and psycho-emotional correlates that imply a greater risk of developing depressive symptoms have not been accurately identified. The purpose of this article, therefore, is to estimate the prevalence of depressive symptoms and to identify the influence of psycho-emotional and contextual stress factors associated with the COVID-19 pandemic on depressive symptoms in men and women in the early months of the pandemic lockdown. We expected to find similar results to those reported in previous research conducted, where the impact on women’s mental health is more severe than on men.

## 2. Materials and Methods

This was an exploratory descriptive study, and data was collected using an online survey; the main objective of the study was to explore substance use and the presence of mental health problems during the lockdown due to the COVID-19 pandemic.

The online survey was conducted using Google Forms in May and June of 2020, the period in which Mexico experienced the strictest lockdown. The link to the questionnaire was published on the official social media accounts (Facebook, (Zuckerberg, Saverin, McCollum, Moskovitz & Hughes, 2004, Cambridge, MA, USA), Twitter, (Dorsey, Williams, Glass & Stone, 2006, San Francisco, CA, USA) and WhatsApp (Acton, Koum, WhatsApp LLC, Menlo Park, CA, USA; Meta Platforms, Inc. version 2.21.15.20, 2009, Cambridge, MA, USA)) of the Ramón de la Fuente Muñiz National Institute of Psychiatry. A total of 4122 individuals were surveyed. All of them were aged 18 years or over, were residents of Mexico, and provided consent for their voluntary participation [33]. The questionnaire comprised 13 sections; however, we have only reported on the following:

Ten questions about sex, age, educational attainment, marital status, occupation, state of origin, income, and family characteristics.

*Adversity and Stress Scale:* Eleven questions formulated for this study were used to measure the stress level caused by the pandemic in different aspects of life during the previous month. The questions were divided into two groups: (a) relational stress, derived from the effects on social interactions at school or work or on the management of free time (six items); and (b) contextual stress, associated with changes in a person’s social and economic status (five items). There were five response options on a Likert scale ranging from 0 (“not at all or only slightly stressful”) to 4 (“very stressful”). The evaluation of the scale’s psychometric characteristics yielded a reliability coefficient of 0.86 for this sample [34].

*Patient Health Questionnaire-2 (PHQ-2)*: This questionnaire included the first two questions from the PHQ-9, which identified depressive symptomatology in the previous two weeks. There were four response options ranging from 0 (“never”) to 3 (“almost every day”) with a maximum possible score of 6 [35]. In Mexico, the discriminating power of this questionnaire has been evaluated with indigenous women, and the best cutoff point found was 3, with a sensitivity of 80% and a specificity of 86.8% [36]. The reliability coefficient for this sample was 0.78. In the meta-analysis by Levis et al. [37], the cut-off point of ≥3 has a 72% sensitivity and 85% specificity independent of the respondent’s sex.

*Perceived threat and experiences with coronavirus:* This was a short version of three scales developed by Conway, Woodard, and Zubrod [38] that explore the perceived threat of the coronavirus (three items, α = 0.89: “Thinking about the coronavirus [COVID-19] makes me feel threatened”, “I am afraid of the coronavirus [COVID-19]”, and “I am stressed around other people because I worry I’ll catch the coronavirus [COVID-19]”), the impact of the coronavirus (six items, α = 0.84: “The coronavirus [COVID-19] has impacted me negatively from a financial point of view”, “I have lost job-related income due to the coronavirus [COVID-19]”, “I have had a hard time getting needed resources (food, toilet paper) due to the coronavirus [COVID-19]”, “It has been difficult for me to get the things I need due to the coronavirus [COVID-19]”, “I have become depressed because of the coronavirus [COVID-19]”, and “the coronavirus (COVID-19) outbreak has impacted my psychological health negatively”), and experiences with coronavirus (seven items, α = 0.71: “I have been diagnosed with the coronavirus [COVID-19]”, “I have had coronavirus-like symptoms at some point in the past two months”, “I have been sick from something other than the coronavirus in the past two months”, “I have been in close proximity with someone who has been diagnosed with coronavirus [COVID-19]”, “I have been in close proximity with someone who has had coronavirus-like symptoms in the last two months”, “I watch a lot of news about the coronavirus [COVID-19]”, and “I spend a huge percentage of my time trying to find updates online or on TV about coronavirus [COVID-19]”). The scales, translated into Spanish for this study, contain seven Likert responses ranging from 1 (“not true of me at all”) to 7 (“very true of me”) [33].

*Emotional state:* This questionnaire was created for the study; it is a list of 12 feelings, consisting of six positive (happiness, hope, pleasantness, joy, relaxation, tranquility) and six negative (boredom, stress, fear, vulnerability, worry, despair) emotions that could be experienced during the lockdown.

Descriptive statistics were used to determine the sociodemographic characteristics of the respondents. The mean score and dimensions of the scales and the prevalence of depressive symptomatology (PHQ-2 score ≥ 3) were obtained. X^2^ and Student’s *t*-tests were performed to evaluate the differences by sex. The effect size was measured with Cramer’s V coefficient for X^2^ tests and Cohen’s d for *t*-tests. Binomial logistic regression models were used to identify the factors associated with depressive symptomatology in men and women. Th adjusted odds ratios (OR) are reported with 95% confidence intervals (CI). The Hosmer–Lemeshow test was used to assess the fit of the models. All statistical analyses were performed using the Statistical Package for the Social Sciences (SPSS) for Windows (version 25.0, IBM Corp., Armonk, NY, USA).

## 3. Results

### 3.1. Respondent Characteristics

A total of 4122 responses were obtained. Only 28.2% of the respondents were men; the majority of respondents were aged 21–40 years (55.9%), were unpartnered (57.1%), had completed undergraduate and postgraduate studies (77.2%), and were employed (69.1%) (for more details about the survey, see Martínez-Vélez et al. [33]).

As can be seen in Table 1, significant differences were found between men and women in the psychosocial factors associated with the pandemic. More women than men reported having previously been in treatment for mental health (29.9% vs. 21.9%) together with a higher percentage of depressive symptoms (38.2% vs. 28.1%) and higher stress scores in interpersonal interactions. Likewise, women reported experiencing negative feelings more often than men and felt more threatened from the coronavirus. Conversely, men reported a greater number of previous chronic diseases than women (34.7% vs. 22.1%) and higher scores on the context-related stress (family economy, family health, socio-economic status); men also reported experiencing more positive feelings with statistically significant differences in relation to women.

### 3.2. Depressive Symptoms

The respondents with depressive symptoms were mostly women, aged 21–30 years, single, and with bachelor’s degrees. People who reported having been in treatment for a mental health problem in the previous 12 months showed the highest percentages of depressive symptoms, and this difference was statically significant; however, the effect size of this difference was low. On the other hand, the lowest scores of depressive symptoms were reported among the group of respondents aged 31–40 years. The highest levels of stress, negative emotions or feelings, impact of coronavirus, experiences with the virus, and feeling threatened by COVID-19 were reported by people with three or more symptoms of depression (Table 2).

### 3.3. Identification of Factors Associated with Depressive Symptoms in Women and Men

The factors associated with depressive symptoms during the COVID-19 lockdown are shown in Table 3. The logistic regression model for men showed that those aged 18–20 years (OR = 2.74, 95% CI [1.33, 5.64]), and those aged 21–30 years (OR = 1.7, 95% CI [1.03, 9.96]) were more likely to present with depressive symptoms than those aged 51 years or over. Those who had a previous history of chronic disease were more likely to have symptoms (OR = 0.69, 95% CI [0.47, 1.48]) than those who did not. Furthermore, those who reported more stress associated with interpersonal relationships (OR = 1.8, 95% CI [1.44, 2.42]), more negative emotions (OR = 1.1, 95% CI [1.13, 1.22]), those experiencing a greater impact of COVID-19 (OR = 1.0, 95% CI [1.01, 1.05]), those with a greater degree of experience of the pandemic (OR = 1.0, 95% CI [1.01, 1.05]), and those with a greater perceived threat from COVID-19 (OR = 0.929, 95% CI [0.981–0.969]) were more likely to present depressive symptoms than those who reported lower levels of these factors.

Younger women were also more likely to present depressive symptoms than women over 51 years old. Having been in mental health treatment in the past 12 months increased the risk of presenting depressive symptoms (OR = 0.77, 95% CI [0.63, 0.95]). Those who reported more stress associated with interpersonal relationships (OR = 1.6, 95% CI [1.40, 1.89]), more negative emotions (OR = 1.1, 95% CI [1.12, 1.17]), and those with a greater impact of the coronavirus (OR = 1.0, 95% CI [1.02, 1.05]) were more likely to present depressive symptoms than those who reported lower levels of these factors.

## 4. Discussion

This study estimated the prevalence of depressive symptoms and their contextual and psycho-emotional correlates among Mexican women and men during the early months of the COVID-19 pandemic lockdown. It was found that 38% of women and 28% of men presented depressive symptoms. The prevalence of these symptoms was much higher than that reported in previous studies in Mexico, which has ranged between 12% and 25% depending on the study population [36,39]; furthermore, it was much higher than the prevalence of major depressive disorder in Mexico, which is estimated to be present in 7.2% of the general population [40]. A similar prevalence of depressive symptoms has been identified as a result of the pandemic in countries such as China and Spain with a greater proportion being found in women than in men [7,8].

Our results suggest that the COVID-19 pandemic has substantially affected the mental health of men and women in Mexico, and these findings are consistent with previous studies that reported that exposure to public health emergencies, such as in the case of the Ebola and SARS outbreaks, can cause mental health problems [41,42]. Global evidence supports the observation of this upward trend in depression symptoms due to emotional distress and stressors related to COVID-19, including the disruption of social relationships, isolation, fears of illness and economic loss, and concern for one’s own health and that of loved ones, all of which can trigger depression or exacerbate existing symptoms [21]. The survey showed high percentages of people who reported that they feared that their mental health would be affected by COVID-19 (between 87% and 92%). There was also a high level of negative emotions experienced during lockdown together with significant stress levels associated with the pandemic.

This high prevalence of stress and negative emotions, especially regarding relationships, was significantly linked to the presence of depressive symptoms for both sexes. These findings coincide with the stress generation model of depression (SGMD), which has documented that both interpersonal and non-interpersonal stress are well-established risk factors for major depressive disorder, with interpersonal stress having the greatest impact [12,19,20]. It has also been observed that positive emotions significantly reduce the effects of interpersonal stress on the severity of depressive symptoms, while negative emotions increase the effects of non-interpersonal stress on their severity [32].

For both men and women, the stressful effect of the COVID-19 pandemic lockdown on areas of life such as personal relationships and the changes in context or life situations led to a situation of continuous tension from different sources, including the economic status and health of the family, which resulted in an increased risk for depressive symptoms [43,44,45].

While the predictors of depressive symptomatology were similar between men and women, there were some differences between the two groups. In general, women tend to experience negative emotions more intensely than men, which can make them more susceptible to depression. The review study of Bracke et al. [46] points to the association between gender inequality and depression, showing that gender differences in depression converge in contexts of greater gender equality and increase in contexts of greater inequality. These effects compound the consequences for the mental health of taking on work and family functions at different stages of life. The SGMD also allows us to understand how gender can shape depression. For example, there is evidence that stress linked to interpersonal relationships can be greater for women, in part because of the greater emphasis such relationships have in women’s lives, especially those characterized by caring for others and emotional closeness [47].

On the other hand, in the case of men, having a pre-existing physical illness and experiencing direct encounters with COVID-19 (experiences of coronavirus) were more associated with depressive symptoms than in women. Rutland-Lawes et al. [48] also identified that a long-standing illness, together with other factors such as alcohol use, living alone/not alone, and employment status were all significant predictors of change in depression scores in men. In this sense, we can hypothesize that the loss of health or functionality and the risk of getting sick could be important predictors of depressive symptoms in men.

The findings of this study allow for a better, timely understanding of the psychological consequences of contingencies such as the COVID-19 pandemic, which is essential for several reasons. First, there is a high prevalence of psychological problems among those directly or indirectly exposed to potentially stressful situations. Such problems can affect the daily functioning of a substantial number of people and can cause immediate social and economic consequences, such as the loss of productivity at work and financial difficulties. Strategies for protecting the psychological health of men and women through mental health interventions are crucial for preventing or offsetting interruptions in the delivery of health services during emergencies.

There is an important need for a greater focus on and understanding of the effect of the environment on depressive disorders in men and women, which is better understood through solid frameworks and hypothetical constructs such as the SGMD. Empirical findings and new perspectives can greatly contribute to our understanding of these phenomena and also to the construction of interventions promoting mental health that are sensitive to differences in gender at times of great stress, such as in the recent pandemic.

*Limitations.* This study has certain limitations. First, its cross-sectional design means that causality cannot be inferred from the results. Second, since the population was under lockdown, the data were collected online through convenience sampling, and the results are therefore not generalizable to the entire population. Third, the answers were self-reported, which may have led to information biases. Finally, the lack of evidence about the validity of the PHQ-2 survey for assessing depressive symptoms among Mexican males is a limitation of our study, although some studies [37] do not make distinctions by gender regarding its sensibility and specificity. Therefore, the results should be taken with caution.

## 5. Conclusions

The findings of this study provide information on the structure and correlates of stress associated with the COVID-19 pandemic and their influence on the presence of depressive symptoms; they add to the knowledge on how socioenvironmental risk influences mental health [49]. The results also shed light on the nature and degree of psychological responses to the pandemic and have the potential to serve as a basis for the development of health promotion and prevention strategies which, together with existing efforts, have the potential to contain the burden of mental illness. The study shows that strategies that promote the mental health of the population should be encouraged to prepare for this type of contingency.

## Figures and Tables

**Table 1:** Percentage distribution of the psychosocial factors associated with the pandemic by sex.

		Men		Women		Total				
		(n = 1160)		(n = 2962)		(n = 4122)				
		*f*	%	*f*	%	*f*	%	X^2^/*df*	*p*	*V* *
**Previous chronic illness**										
	No	757	65.3	2051	69.2	2808	68.1	6.096/1	0.014	0.038
	Yes	403	34.7	911	30.8	1314	31.9			
**Tx mental health in the past 12 months**										
	No	906	78.1	2076	70.1	2982	72.3	26.768/1	0.000	0.081
	Yes	254	21.9	886	29.9	1140	27.7			
**Depressive symptomatology**										
	PHQ2 ≤ 2	834	71.9	1831	61.8	2665	64.7	37.062/1	0.000	0.095
	PHQ2 ≥ 3	326	28.1	1131	38.2	1457	35.3			
		***Mean***	***SD***	***Mean***	***SD***	***Mean***	***SD***	***t/df***	***p***	d **
Relational stress		1.08	0.90	1.30	0.92	1.24	0.92	−6.95/4120	0.000	0.245
Contextual stress		1.44	1.02	1.73	1.04	1.63	1.05	−8.16/4120	0.000	0.285
Positive emotions during lockdown		17.52	5.58	16.92	5.27	17.08	5.36	3.15/4120	0.001	−0.107
Negative emotions during lockdown		16.77	6.46	19.58	6.30	18.79	6.47	−12.79/4120	0.000	0.435
Impact of coronavirus		15.97	8.68	16.22	8.65	16.15	8.66	−0.84/4120	0.401	0.029
Experiences of coronavirus		14.97	7.35	14.75	7.22	14.81	7.25	0.87/4120	0.386	−0.029
Threat of coronavirus		8.63	5.11	9.97	5.61	9.59	5.50	−7.32/4120	0.000	0.261

* Cramer’s *V*; ** Cohen’s d.

**Table 2:** Percentage distribution of the demographic data and psychosocial factors associated with the pandemic due to depressive symptomatology.

		Without Depressive Symptomatology (PHQ2 ≤ 2)		With Depressive Symptomatology (PHQ2 ≥ 3)				
		(n = 2665)		(n = 1457)				
		*f*	%	*f*	%	X^2^/*df*	*p*	*V* *
**Sex**								
	Male	834	31.3	326	22.4	37.062/1	0.000	0.095
	Female	1831	68.7	1131	77.6			
**Age**								
	18–20 years	143	5.4	180	12.4	239.444/4	0.000	0.241
	21–30 years	609	22.9	549	37.7			
	31–40 years	765	28.7	380	26.1			
	41–50 years	600	22.5	218	15.0			
	51 years or over	548	20.6	130	8.9			
**Marital Status**								
	Single	1102	41.4	828	56.8	92.644/3	0.000	0.150
	Married/partnered	1274	47.8	496	34.0			
	Divorced/separated	252	9.5	118	8.1			
	Widowed	37	1.4	15	1.0			
**Education**								
	Elementary/Jr. High	75	2.8	49	3.4	26.799/3	0.000	0.117
	High School	450	16.9	367	25.2			
	Bachelor’s Degree	1413	53.0	756	51.9			
	Graduate Degree	727	27.3	285	19.6			
**Occupation**								
	Homemaker	129	4.8	75	5.1	175.391/5	0.000	0.206
	Unemployed b/l	81	3.0	89	6.1			
	Unemployed s/l	105	3.9	97	6.7			
	Employed	1608	60.3	639	43.9			
	Student	332	12.5	366	25.1			
	Self-employed	410	15.4	191	13.1			
**Previous chronic illness**								
	No	1819	68.3	989	67.9	0.061/1	0.807	0.004
	Yes	846	31.7	468	32.1			
**Mental health Tx in the past 12 months**								
	No	2103	78.9	879	60.3	162.575/1	0.000	0.199
	Yes	562	21.1	578	39.7			
		*Mean*	*SD*	*Mean*	*SD*	***t/df***	***p***	*d* **
Pandemic-related stress								
	Relational stress	0.89	0.74	1.87	0.88	−37.826/4120	0.000	1.323
	Contextual stress	1.32	0.92	2.24	1.00	−29.534/4120	0.000	0.990
Positive emotions during lockdown		18.38	5.23	14.72	4.76	22.143/4120	0.000	−0.699
Negative emotions during lockdown		16.38	5.78	23.21	5.24	−37.462/4120	0.000	1.181
Impact of coronavirus		13.73	7.45	20.57	8.97	−26.149/4120	0.000	0.980
Experiences of coronavirus		13.54	6.54	17.13	7.90	−15.590/4120	0.000	0.547
Threat of coronavirus		8.47	4.99	11.65	5.80	−18.434/4120	0.000	0.636

* Cramer´s *V*; ** Cohen’s d.

**Table 3:** Factors associated with the depressive symptomatology during lockdown by sex.

		Risk of Depressive Symptomatology			
		Men *		Women **	
		(n = 1160)		(n = 2962)	
		OR	*p*	OR	*p*
**Social vulnerability**					
Age					
	51 years or over	1	-	1	
	18–20 years	**2.74**	**0.01**	**4.62**	**0.00**
	21–30 years	**1.72**	**0.05**	**1.90**	**0.00**
	31–40 years	0.96	0.89	1.37	0.07
	41–50 years	0.82	0.52	1.12	0.52
**Psychobiological vulnerability**					
Previous chronic illness					
	No	1	-	1	-
	Yes	**0.63**	**0.01**	0.91	0.37
Tx mental health disorder in the past 12 months					
	No	1	-	1	-
	Yes	0.69	0.06	**0.77**	**0.01**
**Impact of COVID pandemic**					
Pandemic-related stress					
	Relational stress	**1.87**	**0.00**	**1.64**	**0.00**
	Contextual stress	0.95	0.657	1.10	0.18
Positive emotions during lockdown		**0.94**	**0.00**	**0.87**	**0.00**
Negative emotions during lockdown		**1.17**	**0.00**	**1.15**	**0.00**
Impact of coronavirus		**1.04**	**0.00**	**1.03**	**0.00**
Experiences due to coronavirus		**1.03**	**0.02**	1.00	0.68
Threat of coronavirus		**0.93**	**0.00**	0.98	0.08

* Logistic regression model in men (χ^2^ = 460.26, gl = 13, *p* < 0.001); Hosmer–Lemeshow test (χ^2^ = 9.61, gl = 8, *p* = 0.294). The model explained between 33% Cox & Snell and 47% Negelkerke of the variance. The total correct prediction was 81%, and it included 57% of those who had depressive symptoms and 91% of those who did not. ** Logistic regression model in women (χ^2^ = 1296.24, gl = 13, *p* < 0.001); Hosmer–Lemeshow test (χ^2^ = 21.53, gl = 8, *p* = 0.06). The model explained between 35% Cox & Snell and 48% Negelkerke of the variance. The total correct prediction was 80%, and it included 70% of those who had depressive symptoms and 86% of those who did not.

## Data Availability

Not available.
